# Lambeau de couverture au niveau du scarpa par plastie du muscle couturier (Sartorius)

**DOI:** 10.11604/pamj.2015.22.216.7802

**Published:** 2015-11-10

**Authors:** Melek Ben Mrad, Mohammed Ben Hammamia

**Affiliations:** 1 Service de Chirurgie Cardio-vasculaire, Hopital La Rabta, Faculté de Médecine de Tunis, Université Tunis EL Manar, Tunisie

**Keywords:** Lambeau, muscle sartorius, scarpa, flap, sartorius muscle, scarpa

## Image en medicine

L'infection du scarpa avec un pontage prothétique sous-jacent perméable est une urgence chirurgicale qui peut mettre en jeu le pronostic fonctionnel du membre ainsi que le pronostic vital du patient. Le chirurgien vasculaire peut avoir recours dans certaines situations à un traitement conservateur avec débridement local sans explantation du matériel prothétique. Cette option implique la nécessité d'une couverture adéquate du matériel prothétique. La plastie du scarpa par le muscle couturier (sartorius) peut être une bonne alternative. Nous rapportons le cas de monsieur A.M âgé de 60 ans artéritique multi-opéré aux antécédents de plusieurs gestes de revascularisations au niveau des deux membres inférieurs. Le dernier geste de revascularisation a consisté en un pontage axillo-bifémoral. Le patient a été hospitalisé pour une lymphorrhée au niveau du scarpa droit avec à l'examen clinique une prothèse à nu et perméable. Nous avons réalisé un débridement chirurgical, un parage des berges et un lavage abondant au sérum physiologique. Afin de couvrir la prothèse et fermer le scarpa, le muscle sartorius a été disséqué jusqu’à mi-cuisse (A), puis retourné en haut et suturé au scarpa (B). Ce Lambeau, bien vascularisé,a bien permis de bien couvrir la prothèse (C). La peau a été fermée par des points séparés sans tension grâce à des contre incisions de décharge sur la face externe de la cuisse. L’évolution, sous antibiothérapie adaptée, a été favorable sur le plan clinique et biologique avec cicatrisation totale du scarpa (D).

**Figure 1:**
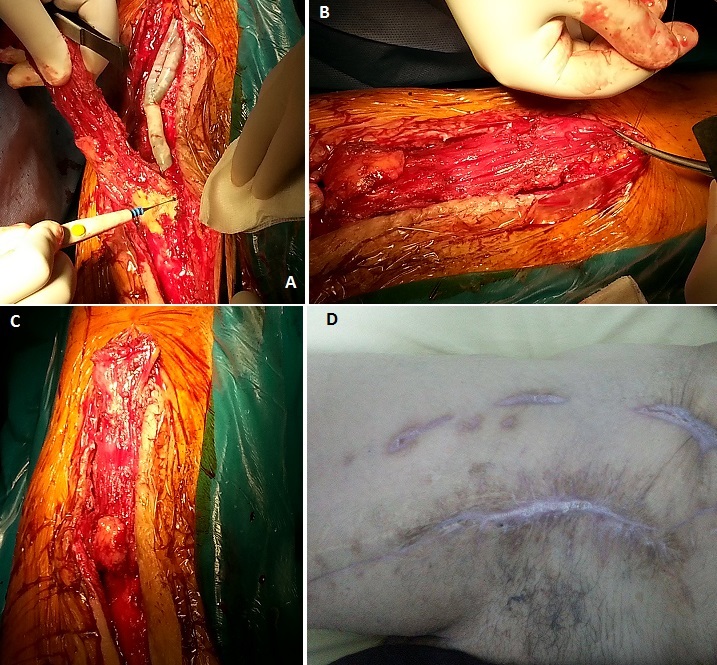
Images peropératoires: A) dissection muscle sartorius; B) suture du muscle sartorius sur le scarpa; C) couverture totale de la prothèse par le lambeau musculaire; D) cicatrisation totale du scarpa

